# Regulation of Adipsin Expression by Endoplasmic Reticulum Stress in Adipocytes

**DOI:** 10.3390/biom10020314

**Published:** 2020-02-17

**Authors:** Ka-Young Ryu, Eon Ju Jeon, Jaechan Leem, Jae-Hyung Park, Hochan Cho

**Affiliations:** 1 Department of Physiology, Keimyung University School of Medicine, Daegu 42601, Korea; rkfud1208@naver.com; 2 Department of Internal Medicine, School of Medicine, Catholic University of Daegu, Daegu 42472, Korea; ejeon@cu.ac.kr; 3 Department of Immunology, School of Medicine, Catholic University of Daegu, Daegu 42472, Korea; 4 Department of Internal Medicine, Keimyung University School of Medicine, Daegu 42601, Korea; ho3632@dsmc.or.kr

**Keywords:** adipsin, endoplasmic reticulum stress, adipocytes, obesity, diabetes mellitus

## Abstract

Adpsin is an adipokine that stimulates insulin secretion from β-cells and improves glucose tolerance. Its expression has been found to be markedly reduced in obese animals. However, it remains unclear what factors lead to downregulation of adipsin in the context of obesity. Endoplasmic reticulum (ER) stress response is activated in various tissues under obesity-related conditions and can induce transcriptional reprogramming. Therefore, we aimed to investigate the relationship between adipsin expression and ER stress in adipose tissues during obesity. We observed that obese mice exhibited decreased levels of adipsin in adipose tissues and serum and increased ER stress markers in adipose tissues compared to lean mice. We also found that ER stress suppressed adipsin expression via adipocytes-intrinsic mechanisms. Moreover, the ER stress-mediated downregulation of adipsin was at least partially attributed to decreased expression of peroxisome proliferator-activated receptor γ (PPARγ), a key transcription factor in the regulation of adipocyte function. Finally, treatment with chemical chaperones recovered the ER stress-mediated downregulation of adipsin and PPARγ in vivo and in vitro. Our findings suggest that activated ER stress in adipose tissues is an important cause of the suppression of adipsin expression in the context of obesity.

## 1. Introduction

The rising prevalence of obesity worldwide and its associated comorbidities, such as type 2 diabetes mellitus (T2DM), has emerged as a major public health challenge [1]. T2DM is characterized by insulin resistance and β-cell dysfunction [2]. Insulin resistance is increased during obesity and initially compensated for by pancreatic β-cells through hypersecretion of insulin. However, when β-cells fail to compensate for the increased insulin demand, T2DM develops. Over the past decades, the underlying mechanisms of β-cell dysfunction have been studied intensively but still remain incompletely understood.

Adipose tissue has been for a long time considered to be no more than a passive tissue for storage of excess energy. However, since the identification of leptin and other adipokines, it became apparent that adipose tissue is a highly active endocrine organ that plays a critical role in regulating systemic energy and glucose homeostasis [3]. Adipsin was first discovered on the basis of the differentiation-dependent expression of its mRNA in cultured adipocytes [4]. It was later identified to be complement factor D, which catalyzes the initial proteolytic step in the alternative complement pathway [5]. Recently, Spiegelman et al. showed that adipsin knockout mice exhibited worsened glucose homeostasis during diet-induced obesity due to insulinopenia [6]. Isolated islets from these mice exhibited decreased insulin secretion. Restoration of adipsin to diabetic mice ameliorated hyperglycemia by augmenting insulin secretion. Given that adipsin expression is markedly decreased in obese animal models and T2DM patients with β-cell failure [6,7,8], these findings suggest that adipsin may be an important link between obesity and β-cell failure. Therefore, identifying the factors that suppress adipsin expression during obesity can help us to develop strategies to restore its expression and prevent or reverse β-cell failure in T2DM.

The endoplasmic reticulum (ER) stress response is an adaptive response used to augment the functional capacity of the ER. It is activated in various tissues under obesity-related conditions [9]. Recent studies have shown that activated ER stress response can induce inflammation and transcriptional reprogramming [9,10]. However, the relationship between adipsin expression and ER stress in adipose tissues during obesity has not yet been elucidated. Therefore, in this study, we aimed to investigate whether ER stress is an underlying cause of the downregulation of adipsin during obesity and explore its molecular mechanisms.

## 2. Materials and Methods

### 2.1. Cell Culture and Treatments

Mouse 3T3-L1 preadipocytes (American Type Culture Collection, Rockville, MD, USA) were grown to confluence in Dulbecco’s Modified Eagle’s Medium (DMEM) containing 10% fetal bovine serum (FBS) at 37 °C under 5% CO_2_ and 95% air. The cells were differentiated into mature adipocytes by culturing them in DMEM containing 10% FBS, 0.5 mM isobutylmethylxanthine, 0.25 μM dexamethasone, and 5 μg/mL insulin. After 2 days, the cells were washed and incubated with DMEM containing 10% FBS and 5 ng/mL insulin for 48 h. To examine the effects of ER stress on adipsin expression, mature 3T3-L1 adipocytes were treated with thapsigargin (0, 1, or 10 nM; Sigma-Aldrich, St. Louis, MO, USA) or tunicamycin (0, 1, or 5 μg/mL; Sigma-Aldrich) for 0, 12, or 24 h. In a separate experiment, after pretreatment with 4-phenylbutyric acid (4-PBA, 10 mM; Sigma-Aldrich) or tauroursodeoxycholic acid (TUDCA, 0.5 mM; Selleckchem, Houston, TX, USA) for 30 min, the cells were incubated with thapsigargin (10 nM) or tunicamycin (5 μg/mL) for 24 h.

### 2.2. Animal Experiments

Seven-week-old male C57BL/6N mice (Central Laboratory Animal Inc., Seoul, Korea) were housed at ambient temperatures (21–23 °C) under a 12:12 h light–dark cycle and had free access to water and food. After a 1 week acclimatization, the mice were randomly divided into two groups fed either a normal chow diet (ND; *n* = 8) or a high-fat diet (60% calories from fat, HFD; *n* = 16). After 8 weeks, the HFD-fed mice were randomly divided into two subgroups: an HFD group (*n* = 8); an HFD plus 4-PBA group (HFD + 4-PBA; *n* = 8). The HFD group and the HFD + 4-PBA group were fed with an HFD and intraperitoneally administered with vehicle (phosphate-buffered saline) or 4-PBA (1 g/kg per day) once daily for additional 4 weeks, respectively. The ND group continued to receive an ND throughout the entire experimental period. After 4 weeks of treatment, all mice were fasted overnight and sacrificed. Blood samples were collected and gonadal white adipose tissues (gWAT) were rapidly isolated for subsequent analyses. To compare expression of adipsin and ER stress markers in gWATs and serum between lean and obese mice, 10-week-old wild-type (WT) C57BL/6N mice (Central Laboratory Animal Inc.) and db/db mice (Central Laboratory Animal Inc.) were sacrificed. To examine the effects of acute ER stress on adipsin expression, vehicle (dimethyl sulfoxide, DMSO), thapsigargin (1 mg/kg), or tunicamycin (2.5 mg/kg) was intraperitoneally injected into C57BL/6N mice. After 6 h, the mice were sacrificed. In addition, to examine the effects of chronic ER stress on adipsin expression, 8-week-old C57BL/6N mice were fed either a ND or an HFD for 12 weeks. All animal experiments were approved by the Keimyung University Institutional Ethics Committee (KM-2018-02).

### 2.3. Biochemical Analysis

Fasting blood glucose levels were measured using the Glucocard Test Strip II (Arkray Inc., Kyoto, Japan). Adipsin levels in mouse serum or culture supernatant were measured using the Mouse Factor D ELISA Kit (Abcam, Cambridge, MA, USA) according to the manufacturer’s instructions.

### 2.4. Quantitative Real-Time Reverse Transcription–Polymerase Chain Reaction (RT-PCR)

Total RNA was extracted from cells or tissues using the TRIzol reagent (Sigma-Aldrich) and then reverse-transcribed into cDNA by using a High-Capacity cDNA Reverse Transcription Kit (Applied Biosystems, Foster City, CA, USA) according to the manufacturer’s instructions. Quantitative real-time RT-PCR was performed using the Power SYBR Green PCR master (Applied Biosystems) and the Real-Time PCR 7500 system (Applied Biosystems). Sequences of specific primers used in this study are listed in Table 1. The relative mRNA levels were calculated by the 2^−ΔΔCt^ method. GAPDH was used as a reference gene.

### 2.5. Western Blot Analysis

Protein samples extracted from cells or adipose tissues were electrophoresed on sodium dodecyl sulfate–polyacrylamide gel electrophoresis (SDS-PAGE) gels and were transferred onto nitrocellulose membranes. The membranes were probed with specific primary antibodies. The anti-adipsin, anti-activating transcription factor 4 (ATF4), anti-C/EBP homologous protein (CHOP), anti-CCAAT/enhancer-binding protein α (C/EBPα), and anti-glyceraldehyde-3-phosphate dehydrogenase (GAPDH) antibodies were purchased from Santa Cruz Biotechnology (Santa Cruz, CA, USA). The anti-glucose-regulated protein 78 (GRP78) and anti-peroxisome proliferator-activated receptor γ (PPARγ) antibodies were purchased from Abcam. The membranes were incubated with horseradish peroxidase-conjugated secondary antibodies. The immunoreactive bands were visualized using enhanced chemiluminescence (Thermo Fisher Scientific, Waltham, MA, USA). GAPDH was used as a loading control.

### 2.6. Small Interfering RNA (siRNA) Transfection

Knockdown of C/EBPα or PPARγ was performed using specific siRNAs (Santa Cruz Biotechnology; sc-37048 and sc-29456, respectively) for each gene. The cells were transfected with 50 nM siRNA using the Lipofectamine RNAiMAX reagent (Thermo Fisher Scientific) according to the manufacturer’s instructions. Scrambled siRNA (Santa Cruz Biotechnology; sc-37007) was used as a negative control for the off-target effects.

### 2.7. Statistical Analysis

Data are represented as the mean ± standard error of the mean (SEM). All statistical analyses were performed using GraphPad Prism 5 (GraphPad Software Inc., San Diego, CA, USA). Differences between groups were analyzed using a Student’s two-tailed t-test or one-way analysis of variance (ANOVA) followed by a post-hoc Bonferroni’s multiple comparison test. All experiments were performed at least 2 times. *p* < 0.05 was considered statistically significant.

## 3. Results

### 3.1. Downregulation of Adipsin is Associated with Increased ER Stress in the Adipose Tissues of Obese Mice

**Figure 1:**
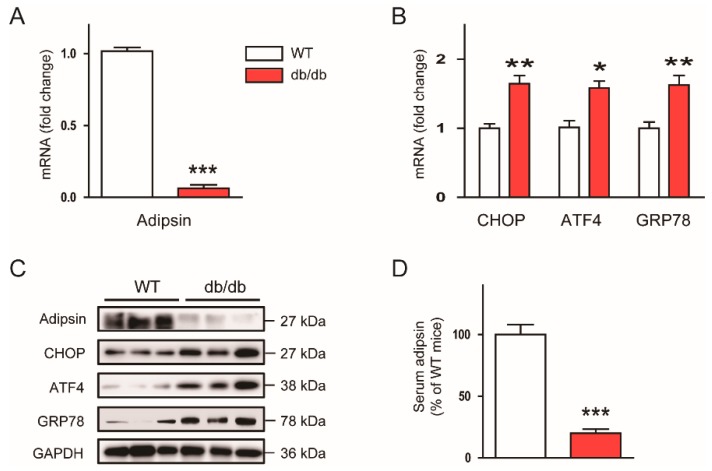
Adipsin expression is inversely associated with endoplasmic reticulum (ER) stress in vivo. To compare expression of adipsin and ER stress markers in gonadal white adipose tissues (gWATs) and serum between lean and obese mice, 10-week-old wild-type (WT) C57BL/6N mice and db/db mice were sacrificed. (**A**) Real-time reverse transcription–polymerase chain reaction (RT-PCR) analysis of adipsin in gWATs. (**B**) Real-time RT-PCR analysis of C/EBP homologous protein (CHOP), activating transcription factor 4 (ATF4), and glucose-regulated protein 78 (GRP78) in gWATs. (**C**) Western blot analysis of adipsin, CHOP, ATF4, GRP78, and glyceraldehyde-3-phosphate dehydrogenase (GAPDH) in gWATs. GAPDH was used as a loading control. Each lane represents an individual animal. (**D**) Serum levels of circulating adipsin. n = 8 per group. All data are represented as the mean ± standard error of the mean (SEM). * *p* < 0.05, ** *p* < 0.01, and *** *p* < 0.001 vs. WT mice.

To investigate the relationship between adipsin expression and ER stress in the context of obesity, we first examined mRNA expression of adipsin and ER stress markers such as CHOP, ATF4, and GRP78 in the adipose tissues (gWATs) of genetically obese db/db mice. We observed that mRNA expression of adipsin was markedly decreased in db/db mice compared to lean WT mice (Figure 1A), whereas mRNA expression of CHOP, ATF4, and GRP78 was significantly increased (Figure 1B). These results were confirmed by western blot analysis (Figure 1C). Besides the expression of adipsin in the adipose tissues, its serum levels were also largely reduced in db/db mice compared to WT mice (Figure 1D). Taken together, these results suggest that adipsin expression is inversely associated with ER stress in obese mice.

### 3.2. ER Stress Suppresses Adipsin Expression in Adipose Tissues

**Figure 2:**
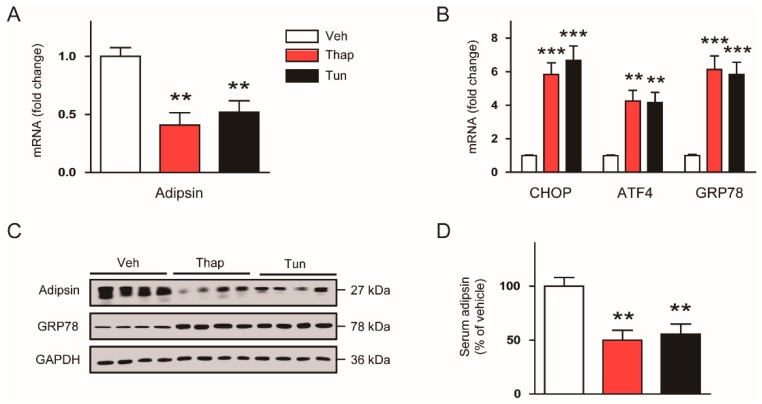
Acute ER stress downregulates adipsin expression in vivo. C57BL/6N mice were intraperitoneally injected with vehicle (Veh; dimethyl sulfoxide), thapsigargin (Thap; 1 mg/kg), or tunicamycin (Tun; 2.5 mg/kg). After 6 h, the mice were sacrificed. (**A**) Real-time RT-PCR analysis of adipsin in gWATs. (**B**) Real-time RT-PCR analysis of CHOP, ATF4, and GRP78 in gWATs. (**C**) Western blot analysis of adipsin, GRP78, and GAPDH in gWATs. GAPDH was used as a loading control. Each lane represents an individual animal. (**D**) Serum levels of circulating adipsin. n = 8 per group. All data are represented as the mean ± SEM. ** *p* < 0.01, and *** *p* < 0.001 vs. Veh-treated group.

Accumulating evidence suggests that activated ER stress response could induce transcriptional reprogramming [9,10], prompting us to further examine whether ER stress can also modulate adipsin expression. We found that expression of adipsin mRNA (Figure 2A) in gWATs of C57BL/6N mice was largely reduced by short-term (6 h) treatment with thapsigargin or tunicamycin, while expression of ER stress markers was increased (Figure 2B). Western blot analysis confirmed the downregulation of adipsin and upregulation of GRP78 induced by the ER stress inducers (Figure 2C). These compounds also markedly decreased serum levels of adipsin (Figure 2D).

**Figure 3:**
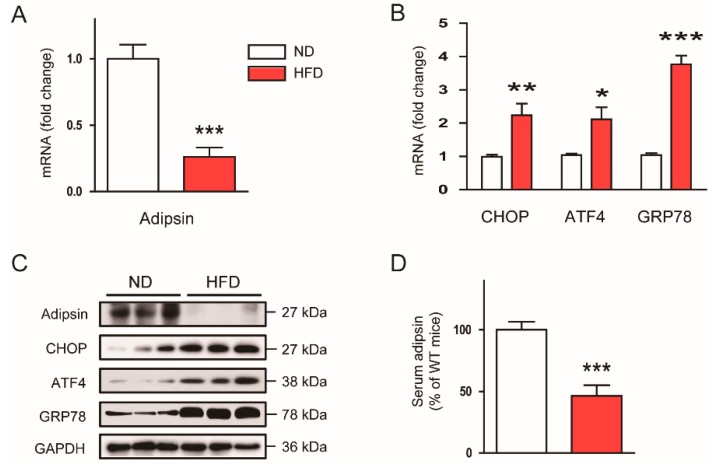
Chronic ER stress downregulates adipsin expression in vivo. C57BL/6N mice were fed a normal chow diet (ND) or a high-fat diet (HFD) for 12 weeks. (**A**) Real-time RT-PCR analysis of adipsin in gWATs. (**B**) Real-time RT-PCR analysis of CHOP, ATF4, and GRP78 in gWATs. (**C**) Western blot analysis of adipsin, CHOP, ATF4, GRP78, and GAPDH in gWATs. GAPDH was used as a loading control. Each lane represents an individual animal. (**D**) Serum levels of circulating adipsin. n = 8 per group. All data are represented as the mean ± SEM. * *p* < 0.05, ** *p* < 0.01, and *** *p* < 0.001 vs. ND-fed group.

To confirm the effect of ER stress on adipsin expression under more physiological conditions, we fed C57BL/6N mice with an HFD for 12 weeks to induce chronic ER stress. We found that mRNA expression of adipsin in gWATs was markedly decreased in HFD-fed mice compared to control ND-fed mice (Figure 3A), while expression of ER stress markers was increased (Figure 3B). These findings were confirmed by western blot analysis (Figure 3C). Serum levels of adipsin were also largely reduced by HFD feeding (Figure 3D). Collectively, these results suggest that acute and chronic ER stress could suppress adipsin expression in mouse adipose tissues.

### 3.3. The ER Stress-Mediated Downregulation of Adipsin is Executed via Intrinsic Mechanisms in Adipocytes

**Figure 4:**
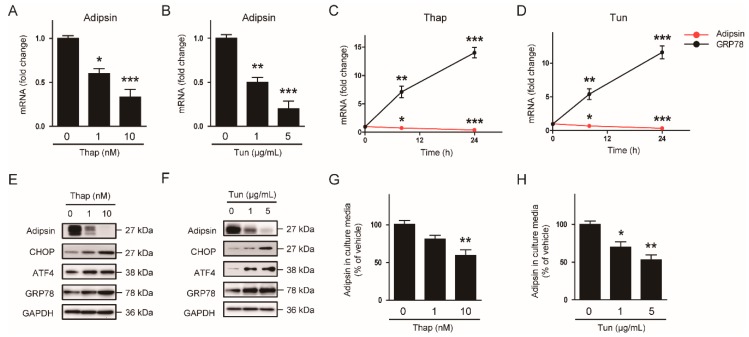
ER stress downregulates adipsin expression in vitro. Mature 3T3-L1 adipocytes were treated with thapsigargin (Thap; 0, 1, or 10 nM) or tunicamycin (Tun; 0, 1, or 5 μg/mL) for 0, 12, or 24 h. (**A**) Dose-dependent effects of Thap on adipsin mRNA at 24 h. (**B**) Dose-dependent effects of Tun on adipsin mRNA at 24 h. (**C**) Time-dependent effects of Thap on mRNA of adipsin and GRP78. (**D**) Time-dependent effects of Tun on mRNA of adipsin and GRP78. (**E**) Dose-dependent effects of Thap on protein levels of adipsin, CHOP, ATF4, GRP78, and GAPDH. GAPDH was used as a loading control. (**F**) Dose-dependent effects of Tun on protein levels of adipsin, CHOP, ATF4, GRP78, and GAPDH. GAPDH was used as a loading control. (**G**) Levels of adipsin secreted into culture media in Thap-treated cells. (**H**) Levels of adipsin secreted into culture media in Tun-treated cells. All data are represented as the mean ± SEM. * *p* < 0.05, ** *p* < 0.01, and *** *p* < 0.001 vs. non-treated cells.

To determine whether ER stress downregulates adipsin in a cell-intrinsic manner, we treated mature 3T3-L1 adipocytes with the ER stress inducers. Thapsigargin (Figure 4A) and tunicamycin (Figure 4B) significantly decreased mRNA expression of adipsin in a dose-dependent manner. In addition, these compounds time-dependently reduced mRNA expression of adipsin, while expression of GRP78 was increased (Figure 4C,D). Protein expression of adipsin or its levels secreted from adipocytes into culture media were also dose-dependently suppressed by thapsigargin (Figure 4E,G) and tunicamycin (Figure 4F,H).

### 3.4. Decreased Expression of PPARγ Contributes to the ER Stress-Mediated Downregulation of Adipsin

**Figure 5:**
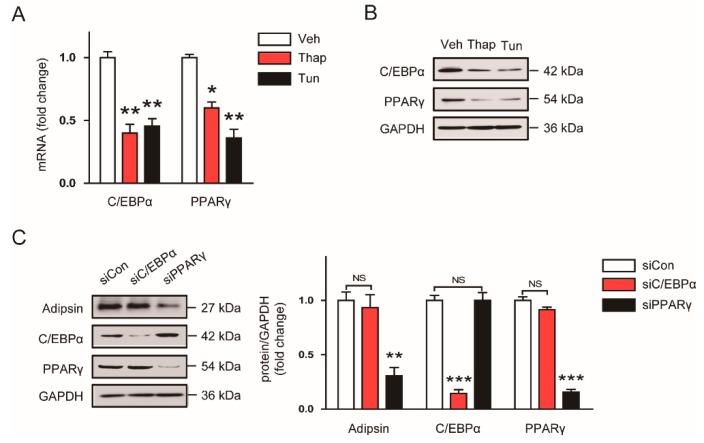
The ER stress-mediated downregulation of adipsin is attributed to reduced expression of peroxisome proliferator-activated receptor γ (PPARγ). (**A**) Mature 3T3-L1 adipocytes were treated with vehicle (Veh; DMSO), thapsigargin (Thap 10 nM), or tunicamycin (Tun; 5 μg/mL) for 24 h. The mRNA levels of CCAAT/enhancer-binding protein α (C/EBPα) and PPARγ were analyzed by real-time RT-PCR analysis. (**B**) Western blot analysis of C/EBPα, PPARγ, and GAPDH in the cells treated with Veh, Thap, or Tun. GAPDH was used as a loading control. (**C**) Knockdown of C/EBPα (siC/EBPα) or PPARγ (siPPARγ) was performed using specific siRNAs for each gene. Scrambled siRNA (real-time) was used as a negative control. The transfected cells were harvested 24 h after transfection, and western blot analysis was performed. The graphs show densitometric quantification of adipsin, C/EBPα, PPARγ normalized against GAPDH. All data are represented as the mean ± SEM. * *p* < 0.05, ** *p* < 0.01, and *** *p* < 0.001 vs. cells treated with Veh or siCon. NS: not significant.

C/EBPα and PPARγ have been implicated as key regulators of adipocyte differentiation and adipokine expression [11,12,13,14]. In addition, previous studies reported that these transcriptional factors were downregulated by ER stress inducers in adipocytes [15,16]. Thus, we hypothesized that downregulation of C/EBPα and PPARγ might play a role in the ER stress-mediated reduction in adipsin expression. We first confirmed that mRNA (Figure 5A) and protein (Figure 5B) expression of C/EBPα and PPARγ were suppressed by treatment with 10 nM thapsigargin or 5 μg/mL tunicamycin. Interestingly, knockdown of PPARγ using siRNA significantly reduced adipsin expression in mature 3T3-L1 adipocytes, while knockdown of C/EBPα did not exhibit significant effect (Figure 5C). Collectively, these results suggest that downregulation of PPARγ by ER stress contributes at least partially to the decreased expression of adipsin in adipocytes.

### 3.5. Chemical Chaperone Recovers the ER Stress-Mediated Downregulation of Adipsin in Vitro and in Vivo

**Figure 6:**
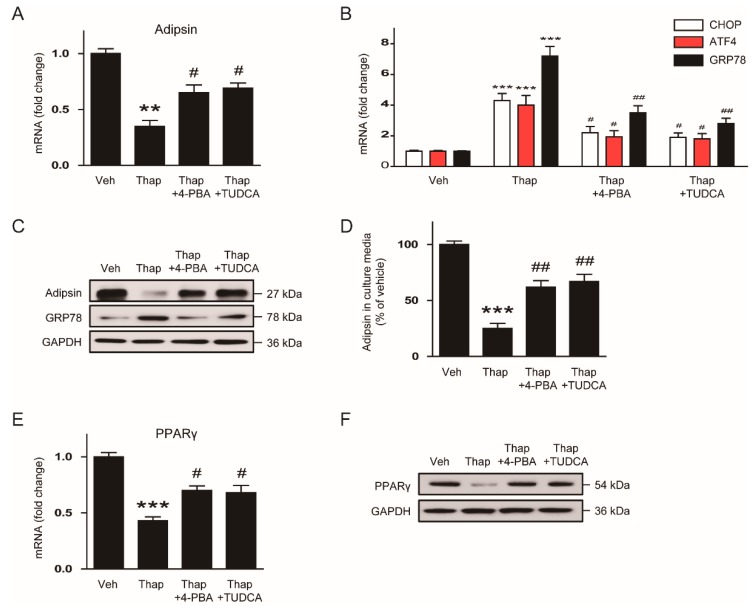
Chemical chaperones reverse the ER stress-mediated downregulation of adipsin in vitro. After pretreatment with 4-phenylbutyric acid (4-PBA; 10mM) or tauroursodeoxycholic acid (TUDCA; 0.5 mM) for 30 min, mature 3T3-L1 adipocytes were incubated with vehicle (Veh; DMSO) or thapsigargin (Thap; 10 nM) for 24 h. (**A**) Real-time RT-PCR analysis of adipsin. (**B**) Real-time RT-PCR analysis of CHOP, ATF, GRP78. (**C**) Western blot analysis of adipsin, GRP78, and GAPDH. GAPDH was used as a loading control. (**D**) Levels of adipsin secreted from the cells into culture media. (**E**) Real-time RT-PCR analysis of PPARγ. (**F**) Western blot analysis of PPARγ and GAPDH. GAPDH was used as a loading control. All data are represented as the mean ± SEM. ** *p* < 0.01 and *** *p* < 0.001 vs. Veh-treated cells. ^#^ *p* < 0.05 and ^##^ *p* < 0.01 vs. Thap-treated cells.

To next examine whether reduced adipsin expression by ER stress is restored by chemical chaperones, mature 3T3-L1 adipocytes were pretreated with 10mM 4-PBA or 0.5 mM TUDCA for 30 min following incubation with 10 nM thapsigargin for an additional 24 h. We found that thapsigargin-induced reduction in adipsin mRNA was significantly restored by the chemical chaperones (Figure 6A), while increased expression of ER stress markers was suppressed (Figure 6B). Western blot analysis also confirmed that the chemical chaperones significantly reversed the downregulation of adipsin and upregulation of GRP78 induced by thapsigargin (Figure 6C). In addition, thapsigargin-induced reduction in levels of adipsin secreted from adipocytes into the culture media was also significantly restored by the chemical chaperones (Figure 6D). Furthermore, we observed that the chemical chaperones significantly reversed thapsigargin-induced reduction in expression of PPARγ mRNA (Figure 6E) and protein (Figure 6F).

**Figure 7:**
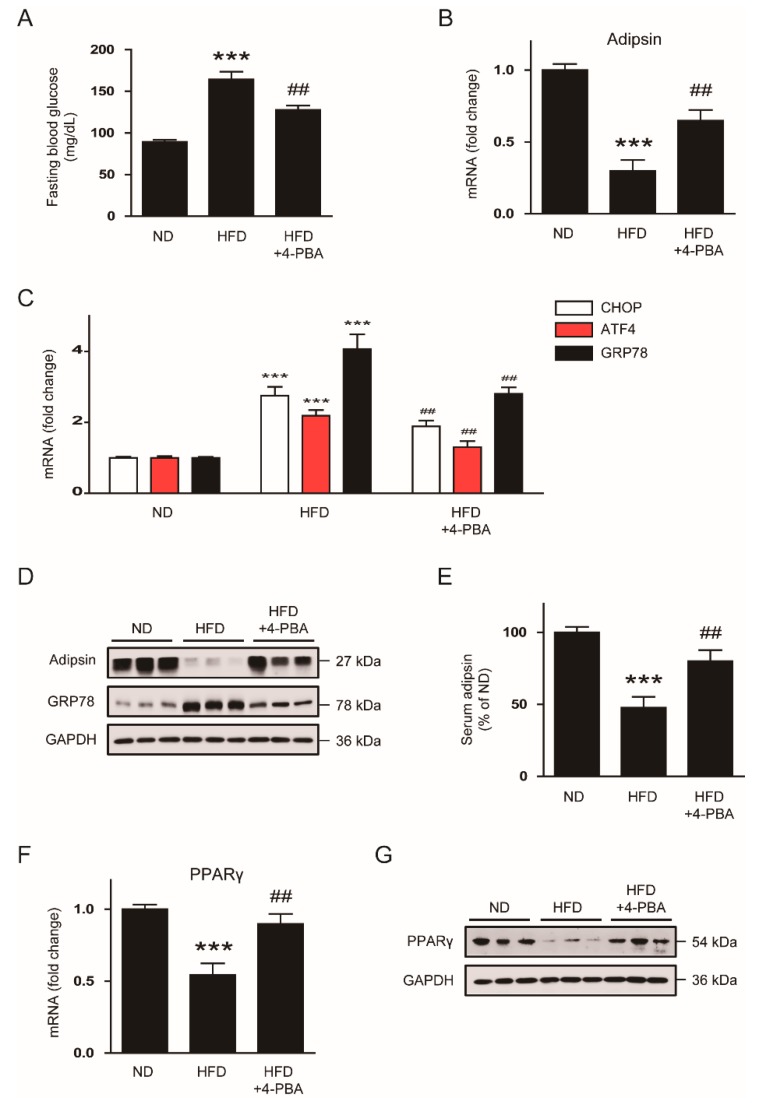
Administration of 4-PBA reverses the ER stress-mediated downregulation of adipsin in vivo. C57BL/6N mice were divided into two groups fed either a normal chow diet (ND; *n* = 8) or a high-fat diet (HFD; *n* = 16). After 8 weeks, the HFD-fed mice were divided into two sub-groups: an HFD group (n = 8); an HFD plus 4-PBA group (HFD + 4-PBA; *n* = 8). The HFD group and the HFD + 4-PBA group were fed with an HFD and intraperitoneally administered with vehicle (phosphate-buffered saline) or 4-PBA (1 g/kg per day) once daily for an additional 4 weeks, respectively. The ND group continued to receive an ND throughout the entire experimental period. After 4 weeks of treatment, all mice were sacrificed. (**A**) Fasting blood glucose levels. (**B**) Real-time RT-PCR analysis of adipsin in gWATs. (**C**) Real-time RT-PCR analysis of CHOP, ATF4, and GRP78 in gWATs. (**D**) Western blot analysis of adipsin, GRP78, and GAPDH in gWATs. GAPDH was used as a loading control. Each lane represents an individual animal. (**E**) Serum levels of circulating adipsin. (**F**) Real-time RT-PCR analysis of PPARγ in gWATs. (**G**) Western blot analysis of PPARγ and GAPDH in gWATs. GAPDH was used as a loading control. Each lane represents an individual animal. All data are represented as the mean ± SEM. *** *p* < 0.001 vs. ND-fed mice. ^##^ *p* < 0.01 vs. HFD-fed mice.

To confirm the effects of the chemical chaperones in obese mice, C57BL/6N mice were fed HFD for 8 weeks following daily intraperitoneal injection of 4-PBA (1g/kg/day) for an additional 4 weeks with HFD feeding. We found that administration of 4-PBA significantly reduced fasting blood glucose levels (Figure 7A) and recovered a reduction in mRNA expression of adipsin in gWATs of HFD-fed mice (Figure 7B), while increased expression of ER stress markers was suppressed (Figure 7C). Administration of 4-PBA also reversed decreased levels of adipsin protein and increased levels of GPR78 protein in gWATs (Figure 7D) and serum (Figure 7E) of HFD-fed mice. Decreased expression of PPARγ mRNA (Figure 7F) and protein (Figure 7G) in gWATs of HFD-fed mice were also largely restored by 4-PBA.

## 4. Discussion

**Figure 8:**
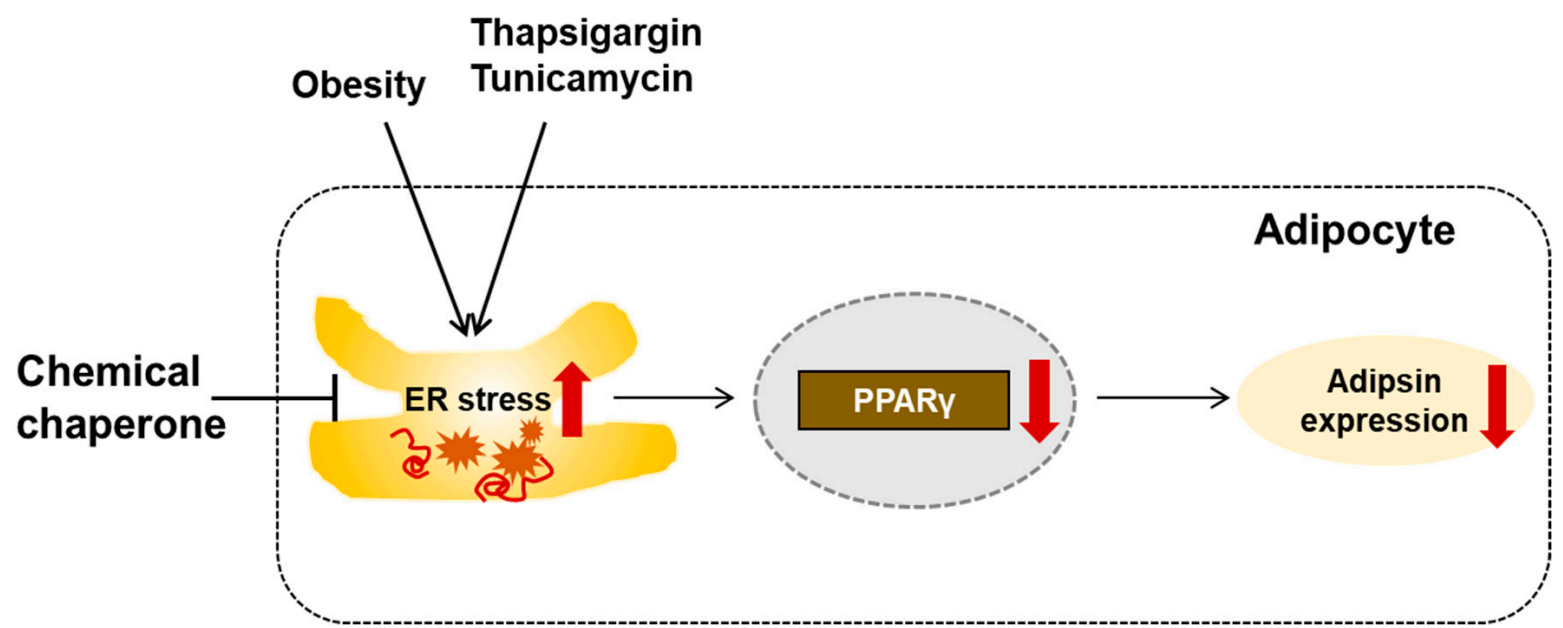
A graphical representation of the results of the present study. Activated ER stress induced by obesity or ER stress inducers (thapsigargin or tunicamycin) reduces adipsin expression, presumably through downregulation of PPARγ in adipocytes. Suppression of ER stress by a chemical chaperone can restore adipsin expression.

In this study, we showed that obesity-induced ER stress downregulates adipsin expression through a transcriptional mechanism in mouse adipocytes. The effects of ER stress on adipsin expression were presumably attributed to downregulation of PPARγ by ER stress. Treatment with chemical chaperones recovered the ER stress-mediated downregulation of adipsin in vitro and in vivo. Taken together, our findings suggest that ER stress activation in adipose tissues of obese mice results in downregulation of adipsin expression, presumably via decreased expression of PPARγ (Figure 8).

The failure of pancreatic β-cell function is one of the key pathogenic processes in the development and progression of T2DM [2]. In the early stages of this disease, β-cells can adequately respond to increased insulin demand through enhancing insulin-secretory capacity. However, as the disease progresses, β-cells fail to secrete sufficient insulin to compensate for insulin resistance. Over the past decades, much effort has been focused on understanding the causes of β-cell failure. Among them, obesity is considered a key risk factor for β-cell failure as well as insulin resistance [2]. Circulating free fatty acid (FFA) levels are elevated in obese individuals, primarily due to a release from increased fat mass. Chronic exposure of pancreatic islets to saturated FFAs induces structural and functional abnormalities, resulting in the development of β-cell failure [17]. However, a molecular link between obesity and β-cell failure still remains incompletely understood.

Recently, Spiegelman et al. demonstrated that adipsin is an adipokine that plays a critical role in maintaining β-cell function [6]. They showed that adipsin knockout mice developed exacerbated diabetes due to β-cell failure, and isolated islets from these mice exhibited decreased insulin secretion. Restoration of adipsin ameliorated hyperglycemia by augmenting insulin secretion in vivo. Furthermore, they identified C3a, a peptide produced by adipsin via alternative complement pathway, as a strong insulin secretagogue and showed that the C3a receptor is required for these favorable effects of the adipokine. The peptide was found to act on islets by increasing ATP levels, respiration, and cytosolic free Ca^2+^. These results revealed the essential role of adipsin in maintaining β-cell function, suggesting that adipsin may be an important link between obesity and β-cell failure. Thus, identifying the factors that suppress adipsin expression during obesity can help us to develop strategies to restore its expression and prevent or reverse β-cell failure in T2DM. However, the pathological causes for inducing downregulation of adipsin have not yet been identified. In the present study, we observed that expression of adipsin mRNA and protein was largely decreased in adipose tissues of genetically obese db/db mice compared to lean WT mice, while expression of ER stress markers such as CHOP, ATF4, and GRP78 was increased, indicating an inverse association between ER stress and adipsin expression. Consistent with our findings, ER stress responses were found to be activated in adipose tissues of obese individuals [18,19]. Activated ER stress response was significantly decreased after gastric bypass surgery with the concomitant reduction in body weight [20]. Gastric sleeve surgery also reduced the body weight and thereby suppressed ER stress in adipose tissues of db/db mice [21]. These findings suggest that obesity induces chronic ER stress in adipose tissues. In addition, previous studies reported that adipsin expression was markedly reduced in obese animal models and T2DM patients with β-cell failure [6,7,8]. Thus, our findings prompted us to examine whether ER stress can suppress adipsin expression. We found that short-term treatment with thapsigargin, a well-known ER stress inducer, markedly reduced expression of adipsin mRNA and protein in adipose tissues of C57BL/6N mice, although it should be considered that the effects of systemic administration of the compound would not be specific to adipose tissues. HFD feeding for 12 weeks also induced downregulation of adipsin with chronic activation of ER stress. Finally, treatment with a chemical chaperone inhibited ER stress and downregulated adipsin in HFD-fed mice. Taken together, these results clearly demonstrate that ER stress suppresses adipsin expression in adipose tissues in the context of obesity.

In the present study, we found that ER stress inducers such as thapsigargin or tunicamycin largely suppressed adipsin expression in mature 3T3-L1 adipocytes, indicating that ER stress downregulates adipsin in a cell-intrinsic manner. However, previous studies have shown that expression of other adipokines, such as adiponectin and resistin, was also reduced by treatment with the ER stress inducers in both adipose tissues and cultured adipocytes [15,22,23]. In addition, expression of C/EBPα and PPARγ was suppressed by the ER stress inducers in mature 3T3-L1 adipocytes [15,16]. C/EBPα and PPARγ are transcription factors that play a key role in the regulation of adipocyte differentiation and adipokine expression [11,12,13,14]. Thus, this may be the mechanism by which ER stress regulates multiple adipokines. In the present study, we hypothesized that downregulation of these transcriptional factors might play a role in the ER stress-mediated reduction in adipsin expression. We first confirmed that ER stress can decrease the expression of C/EBPα and PPARγ in mature 3T3-L1 adipocytes. Interestingly, knockdown of PPARγ using siRNA decreased adipsin expression, while knockdown of C/EBPα did not exhibit significant effect. Taken together, our findings suggest that ER stress-mediated downregulation of PPARγ presumably contributes to a reduction in adipsin expression through a transcriptional mechanism in adipocytes. However, evaluating whether overexpression of PPARγ can restore adipsin expression under ER stress will be required to provide more convincing evidence for the causal involvement of the transcription factor.

The ER is the largest, membrane-bound, intracellular organelle that plays a critical role in protein folding and quality control of newly synthesized proteins. An imbalance between protein load and folding capacity in the ER disrupts homeostasis and causes ER stress, leading to accumulation of misfolded proteins within the lumen of the organelle. ER stress is considered one of the key pathogenic factors in the development and progression of β-cell dysfunction as well as insulin resistance [9]. When the demand for insulin overwhelms the folding capacity of the ER, misfolded proteins are accumulated within the ER lumen of β-cells, inducing β-cell dysfunction and apoptosis. A previous study reported that treatment with 4-PBA ameliorated palmitate-induced β-cell dysfunction [24]. Treatment with TUDCA also prevented β-cell dysfunction and apoptosis in diabetic mice and potentiated glucose-stimulated insulin secretion in isolated mouse pancreatic islets [25,26,27,28]. In the present study, we showed that administration of 4-PBA reduced ER stress and thereby recovered adipsin expression in adipose tissues of HFD-fed obese mice. Moreover, treatment with 4-PBA or TUDCA reversed the ER stress-mediated downregulation in mature adipocytes, although it should be considered that these chemicals can exert other effects besides modulating ER stress. Given that adipsin is an adipokine that enhances β-cell function [6,8], these results suggest adipsin downregulation as a novel mechanism of ER stress-induced β-cell failure. Similar to our findings, recent studies have shown that changes in circulating levels of other adipokines, such as leptin and adiponectin, from adipose tissue affect β-cell function [29]. Taken together, our findings provide evidence that adipsin-mediated crosstalk between adipocytes and β-cells can be modulated by ER stress.

## 5. Conclusions

In conclusion, we demonstrated that activated ER stress suppresses adipsin expression, presumably through downregulation of PPARγ in adipose tissues of obese mice and cultured adipocytes. These results suggest that increasing adipsin expression or inhibiting ER stress in adipose tissues might be potential preventive or therapeutic approaches for β-cell failure.

## Figures and Tables

**Table 1:** Primers for quantitative real-time RT-PCR.

Gene	Primer Sequence (5’→3’)
Adipsin	Forward: CAT GCT CGGCCC TAC ATGG Reverse: CACAGAGTCGTCATCCGTCAC
PPARγ ^1^	Forward: TCGCTGATGCACTGCCTATG Reverse: GAGAGGTCCACAGAGCTGATT
C/EBPα ^2^	Forward: CAAGAACAGCAACGAGTACCG Reverse: GTCACTGGTCAACTCCAGCAC
ATF4 ^3^	Forward: CCTGAACAGCGAAGTGTTGG Reverse: TGGAGAACCCATGAGGTTTCA
CHOP ^4^	Forward: CTGCCTTTCACCTTGGAGAC Reverse: CGTTTCCTGGGGATGAGATA
GRP78 ^5^	Forward: CATGGTTCTCACTAAAATGAAGG Reverse: GCTGGTACAGTAACAACTG
GAPDH ^6^	Forward: ACTCCACTCACGGCAAATTC Reverse: TCTCCATGGTGGTGAAGACA

**^1^** Peroxisome proliferator-activated receptor γ. **^2^** CCAAT/enhancer-binding protein α. **^3^** Activating transcription factor 4. **^4^** C/EBP homologous protein. **^5^** Glucose-regulated protein 78. **^6^** Glyceraldehyde-3-phosphate dehydrogenase.
